# Medical Etiquette

**Published:** 1856-08

**Authors:** G. W. Hall


### IOWA MEDICAL JOURNAL.

VOL. III. KEOKUK, IOWA, JULY AND AUGUST, 1856. NO. 4.

### ORIGINAL COMMUNICATIONS.

### MEDICAL ETIQUETTE--AN ESSAY,

BY G. W. HALL, M. D.,

Before the Hancock County, Medical Society, Illinois.

The medical profession has always been an honorable one. In all ages, the conscientious, upright, and skilful physician has enjoyed the confidence and the esteem of his fellow man. Among the Greeks, medicine was held in so much veneration that Æsculapius was honored with the dignity and adoration of a god. Among the Hebrews, the medical and sacerdotal offices were held by the same chosen priesthood—those who ministered at the Altars of the Jehovah being alone permitted to exercise the functions of the Physician. Among the Hindoos the priest-physician is alone permitted to learn the sacred Sancrit.*

In all ages, and among all people, our profession has been a noble one. We find this the case whether we examine the remotest eras, where fable often usurps the province of authentic history; or the more modern times, where its truth cannot be questioned. Having received such a rich heritage from the past, as this profession of ours, we should endeavor, by every means in our possession, to transmit it untarnished to our posterity. Standing prominently among these means is the subject of Etiquette—the duties which physicians owe to each other, and the respect with which each regards the rights and privileges of his competitors.

*See Renonard's History of Medicine.

Wherever medicine holds the most elevated rank as a science, medical men evince to each other the delicate kindness of brethren having a common object in view. We must respect ourselves, if we would have others respect us. Did every physician duly consider the relation existing between himself and others, and aim to conscientiously observe it, so many ill devised methods would not be resorted to for business, as now are. Unpleasant as it is, perhaps it is proper to mention *some*, among the many means to which, I am sorry to say, too many, claiming to be honorable practitioners, resort to gain practice, and what, in the technical language of such, is termed a "run of business." The most despicable of all is slandering their fellow practitioners; those who have paid more attention than they to the study of the Science of Medicine, and have disdained—to their honor be it said—to try to obtain *tact* in getting customers. How often we hear vague inuendoes, and cowardly slanders started against an honorable physician by his unworthy competitors, in consequence of his having lost a patient, or honestly treated a case in a manner different from theirs. We have enough to do to improve ourselves in professional skill, without having to endure the sly and crafty insinuations of those who, like ourselves, are liable to err every day of their lives. Another violation of the Etiquette of the profession that I will mention is undercharging.

It may be said, however, that a man is the proper judge as to what his services are worth, and is therefore justified in charging as little as he pleases. This may be true, but it is a fact worthy of remembrance by physicians, that the public eventually estimate them by their own standard, and the cheap doctor will be valued according to his own price. Some think they are at liberty to charge but little on the outskirts of their practice, where theirs and another's meet. I know physicians who will ride several miles, and charge a mere nominal sum, to secure the far off business, while near home they charge the same as others. To say the least, this is unfair. It must be a poor consolation to a man to know that he is employed in preference to another, only because he is cheaper in his rates of charging. I envy no one such ill-gotten practice.

Others think that in order to please they must be all things to all men. When with those who believe in the use of calomel, they are also in favor of using it, and when they are with the small pill portion of community, they are opposed to the use of "strong medicine," and claim that they only give such remedies as do no harm if they can do no good, especially to children.

The sooner the people learn, the better for them, that when diseased action exists, it should be removed, even if it require the use of the so-called strong medicine. It is well known that the "strong medicine," when used by skillful hands, will do no harm, and that "mild medicine" used unskilfully, can possibly do no good, and may do harm by not removing morbid action; and this too, with children. This custom is too prevalent, and I trust that a desire to observe a proper etiquette will henceforth deter members of our profession from *practicing* upon the ignorance of people in this way. Consultations, which should only be used for the good of the suffering patient, and for aiding each other, are frequently made the means of gratifying jealousies and petty rivalries. How perverse the man who can seize the opportunity afforded by a consultation and seek thereby to traduce the character and reputation of another. Better had it been if he had never been permitted to occupy a professional position among the illustrious physicians who are honoring the profession with their sterling integrity and upright conduct. It is a fact, the importance of which I wish every physician would recognize, that, by being friendly and honorable towards others, their interests will be promoted pecuniarily, as well as professionally. If selfish motives must solely rule us, it is a mistaken policy to pass our time in devising schemes by which another may be thwarted in his laudable pursuits. I cannot do better here than quote the statement of the venerable Professor Jackson, of Boston, who in a letter to his friend Dr. Warren, published in his "Letters to a Young Physician," after stating that they had always been the warmest friends, like fellow-students all their lives, says, "I would show to them, (the young physicians,) how grateful these results are. I can say to them, our interests have been promoted by our friendly treatment of each other; that each of us has gained by it much more than either of us could have done by the sharpest quarrels. If they believe me, any two of them placed side by side, as we were, may be induced to try the plan of a peaceful competition." Such is the language of one who has long been remarkable for his professional standing and moral worth, and now that his work on earth is nearly done he makes this statement to the young physicians of America. We ought to heed them. Says Dr. Tanner, in his Clinical Medicine, "A man who practices his profession conscientiously will never be unmindful of the duties which he owes to his colleagues,—those treading the same path with himself. He will carefully avoid all such short-sighted proceedings as may tend to elevate himself by depressing others." The most excellent case of Ethics of the American Medical Association, says that "physicians should found their expectations of practice on their qualifications and not on artifice or intrigue."

What a different position our profession would occupy from what it now does if every physician were guided by such sentiments as these! Were this the case, improvements and discoveries in science would no longer be confined to a few, but the whole medical mind of our country could be concentrated on one glorious object—the advancement of science and the amelioration of the condition of man. At no time in the life of a physician is it so important to have proper rules of etiquette fixed indellibly on the mind as in youth, for if he commence his professional career in a wrong manner he is liable to remain so through life. Youth is the time to begin properly, and to lay the foundation for respect and honor when old age shall come. An honorable professional career not only insures peace and quietude here, but it enables the physician to look beyond the grave for his reward. I cannot refrain from quoting here the language of an aged and honorable friend, conveyed to me in a private letter: "Over thirty years," says Professor M'Gugin, "have I been in practice, and thirty-seven a hard student of medicine. Now I am in a condition in which years and time whisper in my ear that my career cannot be long protracted, and seasons and climate, when unfriendly, proclaim the feebleness of my organism. Well, the grave *is* a resting place, and heaven exacts no labor, its climate no sacrifices, and its seasons are without change. There is no chagrin, no false friends, no charlatans, nor rogues, nor scamps there to annoy the pure by their presence, nor the good by their associations." Such is the evidence the honorable physician can have of a life well spent. Professional etiquette requires of us that we should be truthful with each other and with our patients.

Renonard, the author of an excellent history of medicine, recently published in this country, says that "a good practitioner must show himself in all his acts a constant advocate of truth. He must not palliate his reverses or his faults nor exaggerate his success, for he is not ignorant that both serve as lessons, and that in his elevated position to hide the truth or speak falsely is to pave the way for future homicides." How true this is!

Let medical men act from such motives as these; show themselves worthy of their high vocation, of their great scholarship; let them gain knowledge, for it is power, the power that will insure peace and good will here, and peace hereafter.

